# Modulation of Antioxidant Potential with Coenzyme Q10 Suppressed Invasion of Temozolomide-Resistant Rat Glioma *In Vitro* and *In Vivo*

**DOI:** 10.1155/2019/3061607

**Published:** 2019-03-12

**Authors:** Sonja Stojković Burić, Ana Podolski-Renić, Jelena Dinić, Tijana Stanković, Mirna Jovanović, Stefan Hadžić, Jose M. Ayuso, María Virumbrales-Muñoz, Luis J. Fernández, Ignacio Ochoa, Victor M. Pérez-García, Milica Pešić

**Affiliations:** ^1^Department of Neurobiology, Institute for Biological Research “Siniša Stanković”, University of Belgrade, Despota Stefana 142, Belgrade 11060, Serbia; ^2^Group of Applied Mechanics and Bioengineering (AMB), Aragón Institute of Engineering Research (I3A), University of Zaragoza, Zaragoza, Aragon 50018, Spain; ^3^Centro Investigación Biomédica en Red. Bioingenieria, Biomateriales y Nanomedicina (CIBER-BBN), Zaragoza, Aragon 50018, Spain; ^4^Aragon Institute of Biomedical Research, Instituto de Salud Carlos III, Madrid, Madrid 28029, Spain; ^5^Departamento de Matemáticas, E.T.S.I. Industriales and Instituto de Matemática Aplicada a la Ciencia y la Ingeniería (IMACI), Universidad de Castilla-La Mancha, Ciudad Real 13071, Spain

## Abstract

The main reasons for the inefficiency of standard glioblastoma (GBM) therapy are the occurrence of chemoresistance and the invasion of GBM cells into surrounding brain tissues. New therapeutic approaches obstructing these processes may provide substantial survival improvements. The purpose of this study was to assess the potential of lipophilic antioxidant coenzyme Q10 (CoQ10) as a scavenger of reactive oxygen species (ROS) to increase sensitivity to temozolomide (TMZ) and suppress glioma cell invasion. To that end, we used a previously established TMZ-resistant RC6 rat glioma cell line, characterized by increased production of ROS, altered antioxidative capacity, and high invasion potential. CoQ10 in combination with TMZ exerted a synergistic antiproliferative effect. These results were confirmed in a 3D model of microfluidic devices showing that the CoQ10 and TMZ combination is more cytotoxic to RC6 cells than TMZ monotherapy. In addition, cotreatment with TMZ increased expression of mitochondrial antioxidant enzymes in RC6 cells. The anti-invasive potential of the combined treatment was shown by gelatin degradation, Matrigel invasion, and 3D spheroid invasion assays as well as in animal models. Inhibition of MMP9 gene expression as well as decreased N-cadherin and vimentin protein expression implied that CoQ10 can suppress invasiveness and the epithelial to mesenchymal transition in RC6 cells. Therefore, our data provide evidences in favor of CoQ10 supplementation to standard GBM treatment due to its potential to inhibit GBM invasion through modulation of the antioxidant capacity.

## 1. Introduction

Glioblastoma (GBM) is the most common primary malignant tumor with an astrocytic lineage [1]. Treatment options for this type of brain tumor remain limited. The current first-line standard of care is maximal surgical resection and radiotherapy with concomitant and adjuvant chemotherapy with temozolomide (TMZ), a DNA alkylating agent that can cross the blood-brain barrier [2]. Unfortunately, the recurrence rate is high (~ 90%) and the median overall survival of GBM patients is 15 to 18 months, with less than 10% 5-year survival rate [3]. To a vast extent, the reason lies in the high level of intratumor heterogeneity and complex tumor microenvironment resulting in aggressive invasiveness and resistance to radio- and chemotherapy.

Invasion of glioma cells into brain parenchyma is a complex process that includes changes in cell-cell adhesion, remodeling of the extracellular matrix (ECM), and cell migration [4]. Glioma cells have a characteristic migratory pattern along blood vessel membranes or myelinated nerve fibers of the white matter [5]. Cell-cell adhesion is compromised during epithelial-mesenchymal transition (EMT), the process that enables transit of polarized epithelial cells to invasive mesenchymal phenotype. This transition is accompanied by a decreased expression level of epithelial genes (e.g., E-cadherin, ZO-1, and occludin) and increased expression level of mesenchymal genes (e.g., N-cadherin, vimentin, and fibronectin) [6]. Different types of proteases are involved in ECM degradation and remodeling, including members of the matrix metalloproteinase (MMP) family, their inhibitors (tissue inhibitors of metalloproteinases (TIMP)), urokinase-type plasminogen activator (uPA) and its receptor, and cathepsin B. Increased expression of these proteases positively correlates with invasion potential and glioma grade [7, 8]. Malignant gliomas also express a variety of integrin receptors which interact with different components of ECM (e.g., tenascin, laminin, and vitronectin) [9]. This interaction induces cytoskeletal rearrangement and promotes migration. In addition, there are complex bidirectional associations between development of drug resistance and EMT in various types of cancer [10, 11]. Thus, glioma cells resistant to bis-chloroethyl nitrosourea (BCNU), also known as carmustine, showed a significant decrease in E-cadherin expression, increase in vimentin expression and phenotypic changes consistent with EMT, spindle-shaped morphology, and enhanced pseudopodia formation [12].

Resistance to DNA-damaging agents, including TMZ, is usually followed by altered reactive oxygen species (ROS) production in mitochondria [13, 14]. Mitochondria are the major ROS producers due to leakage of electrons from electron transport chains which leads to partial reduction of oxygen and formation of superoxide [15]. Superoxide is dismutated to H_2_O_2_ by manganese superoxide dismutase (MnSOD) in the mitochondrial matrix or copper/zinc SOD (CuZnSOD) in the cytosol. Decomposition of H_2_O_2_ to oxygen and water is mediated by catalase (CAT) and glutathione peroxidase (GPx) with a help of glutathione reductase (GR) [16].

Our RC6 rat glioma model previously established from a C6 cell line is characterized by resistance to BCNU and TMZ (the only two drugs which have been approved so far by FDA for high-grade glioma treatment), lower proliferation rate, and increased invasion potential *in vitro* and *in vivo* [17, 18]. The main mechanism implicated in this resistant and highly invasive phenotype is the alteration of oxidative balance with an elevated level of ROS production and an increased expression of genes involved in redox regulation (*MnSOD*, *GPx*, and *iNOS*) [18].

Herein, we wanted to explore the influence of coenzyme Q10 (CoQ10), a lipid-soluble antioxidant involved in the mitochondrial electron transport chain, on RC6 cell-resistant and invasive phenotypes. In addition to direct antioxidant scavenging, CoQ10 regenerates vitamin E and ascorbate from their oxidized state [19]. CoQ10 supplementation has significant impact on the expression of many genes involved in cell signaling, metabolism, and transport [20]. Various diseases associated with CoQ10 deficiency can benefit from CoQ10 supplementation including cardiovascular and neurodegenerative diseases and cancer [21]. Recently, Frontiñán-Rubio et al. showed that CoQ10 treatment enhances DNA damage induced by radiation and potentiates TMZ cytotoxicity in human glioblastoma cell lines [22]. In addition, some mathematical works have hypothesized a synergistic effect of antioxidants in combination with chemotherapeutics [23].

Therefore, our first goal was to investigate the potential of antioxidant CoQ10 in sensitization of RC6 cells to TMZ, *in vitro* and in microfluidic devices. Next, we explored the single and combined effects of CoQ10 and TMZ on ROS production and expression of enzymatic components involved in redox regulation. We further tested the ability of single and combined CoQ10 and TMZ treatments to suppress the invasive capacity of RC6 cells *in vitro* and *in vivo* and finally examined the mechanism underlying inhibition of RC6 invasive phenotype.

## 2. Materials and Methods

### 2.1. Drugs and Agents

Temozolomide (TMZ) was purchased from Schering-Plough, Labo NV, Heist-op-den-Berg, Belgium. Coenzyme Q10 (CoQ10) (Kaneka Corporation) was kindly provided by Dr. Mario Durán-Prado from the Faculty of Medicine, University of Castilla-La Mancha, Spain. TMZ was diluted in dimethyl sulfoxide (DMSO) and 100 mM aliquots were stored at 4°C. New 2 mM CoQ10 aliquots in DMSO were prepared for each treatment.

### 2.2. Cells and Culture Conditions

RC6 cells were selected from C6 cells by continuous exposure to stepwise increasing concentrations of BCNU for nine months [18]. Cells were maintained in DMEM medium supplemented with 10% FBS, 2 mM L-glutamine, 4.5 g/l glucose, 5000 U/ml penicillin, and 5 mg/ml streptomycin solution at 37°C in humidified 5% CO_2_ atmosphere. Passaging was performed in 72 h intervals using 0.25% trypsin/EDTA, and cells were seeded into fresh medium in a concentration of 8000 cells/cm^2^.

### 2.3. Cell Viability Analysis *In Vitro* and Median-Effect Analysis

Sulforhodamine B (SRB) assay (Sigma-Aldrich, Darmstadt, Germany) was used to assess the combined effect of CoQ10 and TMZ on RC6 cell growth. 2000 cells/well were seeded in 96-well flat-bottom plates and incubated for 24 h at 37°C. Cells were then treated for 72 h with different concentrations of TMZ (10, 25, 50, 100, and 250 *μ*M) combined with different concentrations of CoQ10 (5, 10, and 25 *μ*M). Afterwards, cells were fixed in 50% trichloracetic acid for 1 h at 4°C, rinsed in tap water, and stained with 0.4% SRB in 1% acetic acid for 30 min at room temperature. The cells were then rinsed three times in 1% acetic acid to remove the unbound stain. The protein-bound SRB stain was extracted with a 10 mM Tris base. Absorbance was measured at 540 nm using an automated microplate reader (LKB 5060-006 Microplate Reader, LKB, Vienna, Austria). Half-maximal inhibitory concentration (IC50) values were defined as the concentration of the drug that inhibited cell growth by 50% and was calculated by nonlinear regression analysis using GraphPad Prism 6 software (La Jolla, CA, USA).

The median-effect analyses are based on the median-effect principle established by Chou and Talalay [24] which was used to calculate the combination index (CI) value for the interaction between TMZ and CoQ10. The analyses were carried out using CalcuSyn software (Biosoft, Cambridge, UK). We used at least three data points for each single drug in each designed experiment. The nonconstant ratio combination was chosen to determine the effect of both drugs in combination. CI < 1 indicates synergism, CI = 1 indicates an additive effect, and CI > 1 indicates antagonism.

### 2.4. Cell Viability Analysis in Microfluidic Devices

The microfluidic devices used in our experiments were designed by the Group of Applied Mechanics and Bioengineering (AMB), Aragón Institute of Engineering Research (I3A), University of Zaragoza, Spain, and fabricated by BEONCHIP (Spain). The microfluidic chips were previously validated in drug toxicity analysis [24]. The microfluidic device comprises of a central microchamber and two lateral microchannels. Due to its specific design, a hydrogel can be confined in the central microchamber without invading the lateral microchannels. In this way, a 3D system was developed with the tumor cells being embedded in a collagen hydrogel, mimicking extracellular matrix, in the central microchamber. The lateral microchannels remained hydrogel-free, mimicking blood vessels, and were used for the delivery of medium with nutrients, oxygen, or drugs as required.

For the preparation of three-dimensional cell cultures in collagen hydrogels, all reagents and microdevices were placed on ice. 4 × 10^6^ RC6 cells were resuspended in 50 *μ*l of medium. Cell suspension was then mixed with 50 *μ*l of hydrogel (35.7 *μ*l of collagen type I (Corning®, New York, USA); 0.89 *μ*l 1 M NaOH; 10 *μ*l 5x DMEM; 3.4 *μ*l dH_2_O), and 10 *μ*l of this mixture was injected in the central microchamber. Polymerization of the hydrogel was carried out for 15 min in the incubator, and then, lateral microchannels were perfused with medium to allow oxygen and nutrient diffusion. After 2 h, this medium was removed and a fresh one with 10 *μ*M CoQ10, 250 *μ*M TMZ, or their combination was perfused through the lateral microchannels. Drops of medium were left at each lateral microchannel inlet to prevent evaporation. Cell viability was examined after 72 h with calcein (CAM) (1 : 1000; Thermo Fisher Scientific, MA, USA)/propidium iodide (PI) (4 *μ*g/ml; Sigma-Aldrich, Darmstadt, Germany) staining. Images of microdevices were taken using a Nikon Eclipse Ti microscope (Nikon Instruments Inc., Tokyo, Japan) equipped with a C1 modular confocal microscope system, at 2x magnification. The results were analyzed using Fiji® software and represented as PI fluorescence intensity across the microdevice. At least three independent experiments were performed.

### 2.5. ROS Production in Microfluidic Devices

Preparation of three-dimensional cell cultures in microfluidic devices was performed as described in Cell viability analysis in microfluidic device subsection. The medium with 10 *μ*M CoQ10 was perfused through the lateral microchannels. ROS production was examined using 25 *μ*M CellROX® Orange (Thermo Fisher Scientific, MA, USA) that was also perfused through the lateral microchannels after 2 h and 4 h. Images of microdevices were taken using a Nikon Eclipse Ti microscope equipped with a C1 modular confocal microscope system, at 10x magnification. The results were analyzed using Fiji® software and represented as fluorescence intensity of oxidized CellROX® Orange across the segment of the microdevice. At least three independent experiments were performed.

### 2.6. RNA Extraction, Reverse Transcription, and Real-Time Quantitative PCR

RC6 cells were treated with 10 *μ*M CoQ10, 250 *μ*M TMZ, or their combination for 24 h. Total RNA was isolated from these samples and control RC6 cells without treatment using TRIzol® reagent (Invitrogen Life Technologies, MA, USA) according to the manufacturer's instructions. RNA was quantified by spectrophotometry, and quality was determined by agarose gel electrophoresis. RT reactions were performed using 2 *μ*g of total RNA, with a high-capacity cDNA reverse transcription kit (Applied Biosystems, CA, USA), following the manufacturer's instructions.

Real-time quantitative PCR (qRT-PCR) was performed in order to determine MnSOD [25], CuZnSOD, CAT, GR [26], iNOS [27], MMP9 [17], and *β*-actin [28] gene expression levels. Prepared 100 ng cDNAs were amplified using Maxima SYBR Green/ROX qPCR Master Mix (Thermo Fisher Scientific, MA, USA), according to the recommendations of the manufacturer, in a QuantStudio 3 Real-Time PCR System (Thermo Fisher Scientific, MA, USA). Thermocycler conditions comprised an initial step at 50°C for 5 min, followed by a step at 95°C for 10 min and a subsequent 2-step PCR program at 95°C for 15 s and 60°C for 60 s for 40 cycles. The accumulation of PCR products was detected in real time; the results were analyzed using the QuantStudio™ Design and Analysis Software 1.3.1. and presented as 2^−ΔCt^ [29], ΔCt being the difference between Ct values of specific genes and *β*-actin as endogenous control.

### 2.7. Protein Isolation and Western Blot Analysis

RC6 cells were treated with 10 *μ*M CoQ10, 250 *μ*M TMZ, or their combination for 24 h. Proteins were isolated from these samples and control RC6 cells without treatment in RIPA buffer containing complete protease and phosphatase inhibitor cocktails (Roche, Mannheim, Germany). Total protein concentrations were measured with Micro BCA™ Protein Assay Kit (Thermo Fisher Scientific, MA, USA), using bovine serum albumin as standard.

Equal amounts of proteins (35 *μ*g) were separated by 8% or 12% SDS-PAGE and transferred onto PVDF membranes (GE Healthcare, Buckinghamshire, UK). The membranes were blocked with 3% bovine serum albumin in blotto base buffer (0.1% Tween 20, 20 mM Tris-HCl pH 7.6, 137 mM NaCl) for 1 h at room temperature and incubated overnight at 4°C in the same buffer containing rabbit polyclonal antibodies specific to CAT (1 : 2000; ab16731; Abcam, UK), GR (1 : 2000; ab16801; Abcam, UK), CuZnSOD (1 : 2000; ab13533; Abcam, UK), MnSOD (1 : 5000; ab13498; Abcam, UK), iNOS (1 : 1000; MABN527; Darmstadt, Germany), or GAPDH (1 : 5000; G9545; Darmstadt, Germany) and mouse polyclonal antibodies specific to vimentin (1 : 1000; M0725; Dako, Denmark) or N-cadherin (1 : 5000; 610921; BD Biosciences, USA). After washing for five times with blotto base buffer, horseradish peroxidase- (HRP-) conjugated bovine anti-rabbit IgG secondary antibody (1 : 5000; ab6721; Abcam, UK) or rabbit anti-mouse IgG secondary antibody (1 : 3000; P0260; Dako, Denmark) was applied in the same buffer for 1 h at room temperature. Immunoreactive bands were detected on medical X-ray blue film (Carestream Health Inc., NY, USA) by the enhanced chemiluminescence (ECL) detection system (Santa Cruz Biotechnology, TX, USA) according to the manufacturer's instructions. Densitometric quantification of immunoreactive bands was performed using ImageQuant (GE Healthcare) image analysis software (ver. 5.2) and expressed as relative values normalized to GAPDH as internal control.

### 2.8. Gelatin Degradation Assay

RC6 cells were plated on top of glass coverslips (20000 cells per coverslip) coated with Alexa Fluor 488-labeled gelatin (Gelatin from Pig Skin, Oregon Green® 488 Conjugate, Life Technologies, MA, USA) in 6-well plates. The cells were treated with 10 *μ*M CoQ10, 250 *μ*M TMZ, or their combination. Corresponding control was used. After 24 h, cells were fixed with 4% paraformaldehyde (PFA) and costained with Hoechst 33342 (1 : 1000; Sigma-Aldrich, Darmstadt, Germany) and ActinRed 555 (1 : 500; Invitrogen Life Technologies, MA, USA). The coverslips were analyzed at a 20x magnification under a Zeiss Axiovert inverted fluorescent microscope (Carl Zeiss Foundation, Heidenheim, Germany). The volume of the dark area caused by degradation of gelatin was measured in ImageJ software (v.1.48, Microsoft, WA, USA) and normalized in relation to the volume of the cell. At least 100 cells were analyzed per experiment.

### 2.9. Matrigel Invasion Assay

In Matrigel invasion assay, Transwell inserts (membrane pore size, 8 *μ*m; diameter, 6.4 mm; BD Biosciences Discovery Labware, USA) were placed in 24-well plates. 70000 RC6 cells were seeded in serum-free medium in the upper chambers covered with a layer of Matrigel Basement Membrane Matrix (5 mg/ml, BD Biosciences) and treated 24 h with 10 *μ*M CoQ10, 250 *μ*M TMZ, or their combination. Corresponding untreated cells were used as a positive control. The lower chambers were filled with DMEM medium supplemented with 10% FBS as chemoattractant. A negative control with serum-free medium in the lower chamber was also included in the experiment. After the incubation, cells that invaded through the Matrigel and its underlying membrane were fixed in 4% PFA and stained with Hoechst 33342 (1 : 1000). Cells (their nuclei) present at the lower surfaces of the membranes were counted under a Zeiss Axiovert inverted fluorescent microscope, at 10x magnification. The average number of cells in 30 independent fields per membrane was analyzed using ImageJ software. At least three independent experiments were performed.

### 2.10. 3D Tumor Spheroid Invasion Assay

RC6 spheroids were generated using the hanging drop method with methylcellulose, as previously described [17]. For 3D invasion assay, 25 *μ*l of spheroids was embedded in hydrogel mixture (17.85 *μ*l collagen type I; 0.45 *μ*l 1 M NaOH; 5 *μ*l 5x DMEM; 1.7 *μ*l dH_2_O), placed on top of 50 *μ*l of hydrogel, and treated with 10 *μ*M CoQ10, 250 *μ*M TMZ, their combination, or 50 *μ*M GM6001 (Selleckchem, TX, USA). Control without treatment was included. After 24 h, spheroids were stained with CAM (1 : 1000) and PI (1 : 500) in order to visualize spheroids and test viability. Images of spheroids were taken using a Nikon Eclipse Ti microscope equipped with a C1 modular confocal microscope system, at 20x magnification. The size of spheroids was analyzed using Fiji® software. At least 10 spheroids were analyzed per experiment.

### 2.11. Animal Studies

Three-month-old male Wistar rats were used for *in vivo* experiments. All animal procedures were in compliance with Directive (2010/63/EU) on the protection of animals used for experimental and other scientific purposes and were approved by the Ethical Committee for the Use of Laboratory Animals of the Institute for Biological Research “Siniša Stanković,” University of Belgrade. The animals were housed under standard conditions (23 ± 2°C, 60%–70% relative humidity, 12 h light and dark cycles, and free access to water and food).

The animal model was established as previously described by Stojković et al. [17]. Briefly, animals were anesthetized with Nembutal (Serva, Heidelberg, Germany) at 50 mg/kg, administered intraperitoneally (i.p.). 10^5^ CFSE-labeled RC6 cells (Invitrogen Life Technologies, MA, USA) in 5 *μ*l suspension were injected in brain parenchyma (primary motor cortex) using a Hamilton syringe. TMZ and CoQ10 were dissolved in DMSO and further diluted in 0.9% NaCl for subsequent i.p. administration. Animals were randomly divided into 4 groups; each group consisted of 5 animals: the control group, the group that received 10 mg/kg CoQ10 (starting the 5th day after cell inoculation, 2 times a week for three weeks), the group that received 4 mg/kg TMZ (starting the 12th day after cell inoculation, 5 days consecutively), and the CoQ10 + TMZ group that received the combination regimen. After 25 days, animals were sacrificed and brains were removed and fixed in 4% PFA and cryoprotected in 30% sucrose in PBS. The brains were cut in coronal sections and stained with Hoechst 33342 (1 : 1000) for nuclei visualization. Images of coronal sections were taken using a Zeiss Axiovert inverted fluorescent microscope, at 5x magnification.

### 2.12. Statistical Analysis

Statistical analyses were performed by GraphPad Prism 6 software (La Jolla, CA, USA). Data normality was estimated using Shapiro-Wilk's test. The data obtained by SRB assay were analyzed by two-way ANOVA, using Dunnett's multiple comparisons test. Invasion assays, qRT-PCR, and Western blot experiments were analyzed by Student's *t*-test. Gelatin degradation data did not have a normal distribution, so the Wilcoxon matched-pair signed rank test was carried out. The observed differences were considered statistically significant if *p* < 0.05.

## 3. Results

### 3.1. CoQ10-Sensitized RC6 Cells to TMZ *In Vitro* and in Microfluidic Devices

**Figure 1:**
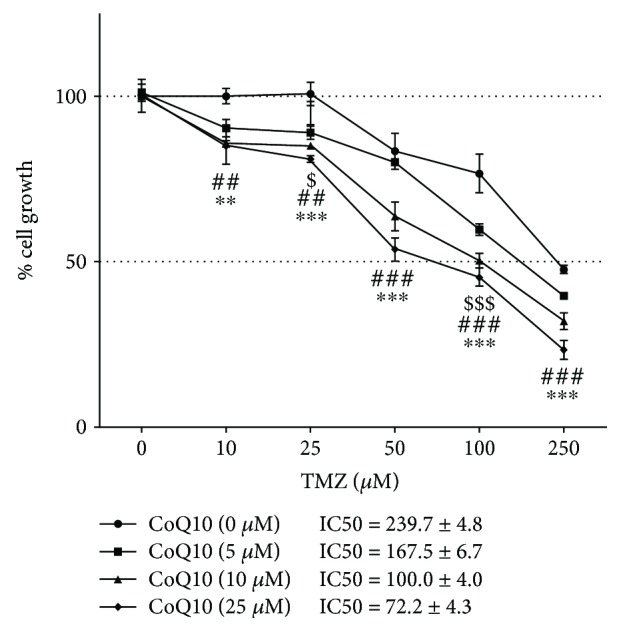
Combined effect of CoQ10 and TMZ on RC6 cell growth. Cell viability was determined using SRB assay 72 h after treatment. IC50 values for each CoQ10 concentration are included next to the graph. The average ±SEM was obtained from four independent experiments. Statistical significance: 5 *μ*M CoQ10 to 0 *μ*M CoQ10, *p* < 0.05 ($) and *p* < 0.001 ($$$); 10 *μ*M CoQ10 to 0 *μ*M CoQ10, *p* < 0.01 (##) and *p* < 0.001 (###); 25 *μ*M CoQ10 to 0 *μ*M CoQ10, *p* < 0.01 (∗∗) and *p* < 0.001 (∗∗∗).

First, we assessed the inhibitory effect of combined CoQ10 and TMZ treatments on RC6 cell growth after 72 h using the SRB assay. The application of all CoQ10 concentrations (5 *μ*M, 10 *μ*M, and 25 *μ*M) decreased the IC50 values for TMZ. More prominent effects were obtained with 10 *μ*M and 25 *μ*M CoQ10 concentrations, showing 2.4-fold and 3.3-fold decreases in IC50 for TMZ, respectively (Figure 1). The obtained results were analyzed using a synergism/antagonism CalcuSyn software tool. Concentrations of 10 *μ*M CoQ10 and 250 *μ*M TMZ whose combination index (CI) was 0.427 were used in single and combined treatments in the following experiments. Nevertheless, all tested combinations showed synergistic effects having CI < 1 (data not shown).

**Figure 2:**
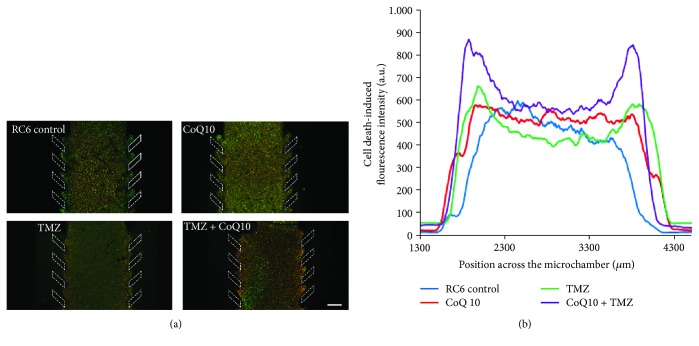
Cell death induction in a microfluidic device. (a) Representative images of microfluidic devices with RC6 cells embedded in collagen hydrogel in the central microchamber. Growth medium and medium supplemented with 10 *μ*M CoQ10, 250 *μ*M TMZ, or their combination were perfused through both lateral microchannels, and after 72 h, cell viability was assessed by CAM (green) and PI (red) staining. The position of the pillars is defined with white dashed lines. Scale bar = 500 *μ*m; (b) the graph displays the PI fluorescence intensity along the delimited region in control, single, and combined CoQ10 and TMZ treatments.

Then, microfluidic devices were employed to study the efficacy of the proposed treatments in conditions that reflect more accurately the complex tumor microenvironment. 4 × 10^7^ RC6 cells/ml were embedded in a collagen hydrogel and injected in the central microchamber of microfluidic device. Lateral microchannels were perfused with growth medium, or medium supplemented with 10 *μ*M CoQ10, 250 *μ*M TMZ, or their combination. After 72 h, cells were stained with CAM and PI (Figure 2(a)). Treatment with CoQ10 did not change the intensity of PI fluorescence implying that CoQ10 did not induce cell death. TMZ treatment led to an increase of the PI fluorescence intensity in an area adjacent to the lateral microchannels confirming its cytotoxicity. Combined treatment produced more intense increase of PI fluorescence in a much wider area adjacent to the lateral microchannels compared to single TMZ treatment suggesting synergistic interaction between two drugs (Figure 2(b)).

### 3.2. CoQ10, Alone and in Combination with TMZ, Modulated Oxidative Balance and Antioxidant Enzyme Expression in RC6 Cells

**Figure 3:**
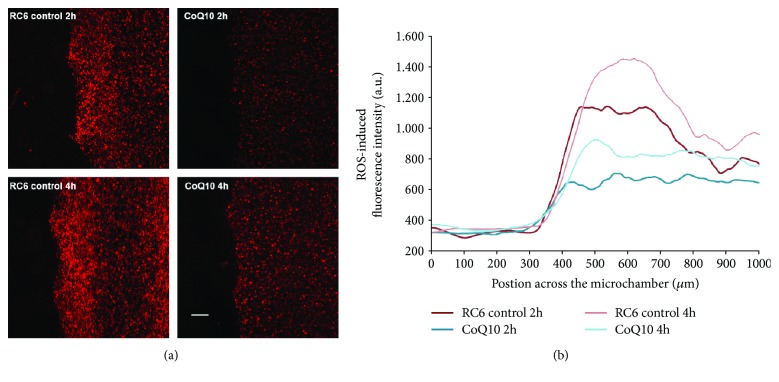
ROS production in a microfluidic device. (a) Representative images of microfluidic devices with RC6 cells embedded in collagen hydrogel in the central microchamber. Free medium or medium with 10 *μ*M CoQ10 was perfused through both lateral microchannels; after 2 h and 4 h, cellular ROS production was assessed by CellROX® Orange. ROS oxidizes CellROX® Orange to fluorescent product (red). Scale bar = 200 *μ*m. (b) The graph displays the oxidized CellROX® Orange fluorescence intensity along the delimited region in control and CoQ10 treatment.

Microfluidic devices were also used to assess the effect of CoQ10 on ROS production in RC6 cells. Again, 4 × 10^7^ RC6 cells/ml were embedded in collagen hydrogel and injected in the central microchamber. Lateral microchannels were perfused with growth medium or medium supplemented with 10 *μ*M CoQ10. After 2 h and 4 h, cells were stained with CellROX® Orange (Figure 3(a)). Results are presented as intensity of fluorescence that corresponds to oxidized reagent CellROX® Orange (ROS indicator). Treatment with CoQ10 led to decrease in ROS production at both tested time points (Figure 3(b)). The effect did not fade from 2 h to 4 h indicating that ROS decrease after CoQ10 application is not transient.

**Figure 4:**
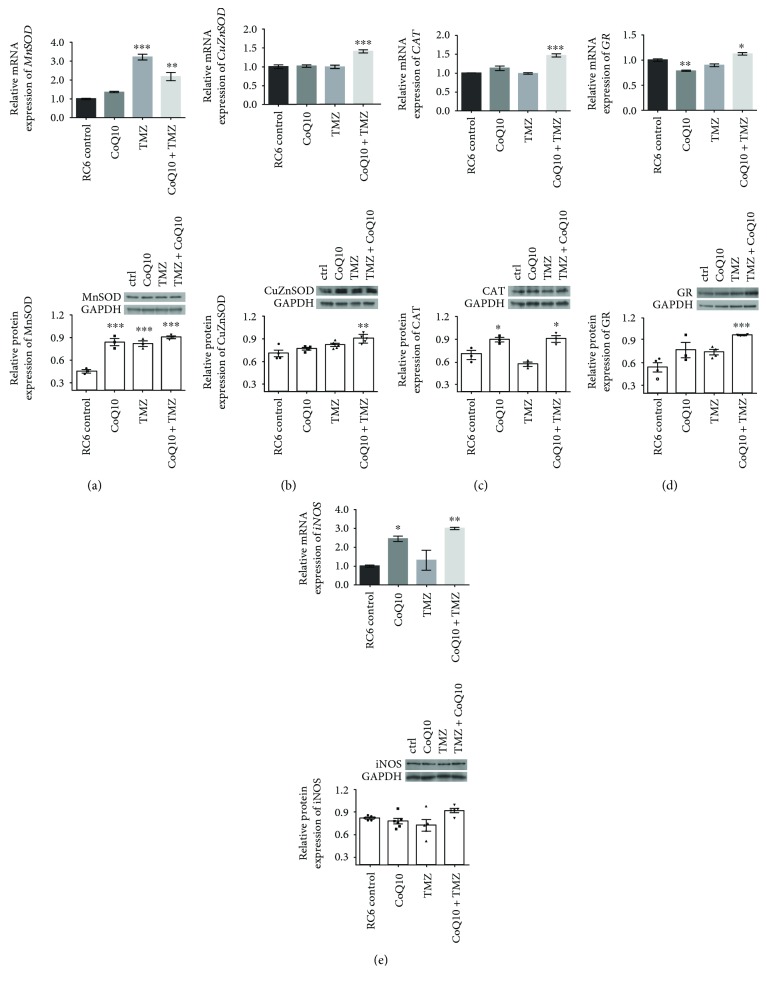
Effects of single and combined CoQ10 and TMZ treatments on gene and protein expression of antioxidant enzymes. Real-time qRT-PCR and Western blot analysis were used to assess (a) MnSOD, (b) CuZnSOD, (c) CAT, (d) GR, and (e) iNOS mRNA and protein expression after 24 h treatment with 10 *μ*M CoQ10, 250 *μ*M TMZ, or their combination. The mRNA expression and protein expression were normalized to the internal control, *β*-actin and GAPDH, respectively. Relative protein expression is accompanied by representative immunoblots. The average ±SEM was obtained from at least three independent experiments. Statistical significance is presented as *p* < 0.05 (∗), *p* < 0.01 (∗∗), and *p* < 0.001 (∗∗∗).

To test if any components of the antioxidative system could be modulated by CoQ10, we analyzed the expression of MnSOD, CuZnSOD, CAT, GR, and iNOS by qRT-PCR and Western blot in RC6 cells treated for 24 h with 10 *μ*M CoQ10 and 250 *μ*M TMZ, alone and in combination (Figure 4). Treatments with TMZ alone and in combination with CoQ10 led to a significant increase of *MnSOD* mRNA expression in RC6 cells of 3.2-fold (*p* ≤ 0.001) and 2.2-fold (*p* ≤ 0.01), respectively. This trend was also detected at a protein level, where all three treatments led to a similar increase, around 2-fold (*p* ≤ 0.001), in protein expression (Figure 4(a)). The combination also increased mRNA expression and protein expression of CuZnSOD by 1.4-fold (*p* ≤ 0.001) and 1.3-fold (*p* ≤ 0.01), respectively (Figure 4(b)). Increased *CAT* mRNA expression by the combined treatment (1.5-fold, *p* ≤ 0.001) was followed by a similar increase in CAT protein expression (1.3-fold, *p* ≤ 0.05) (Figure 4(c)). Additionally, cotreatment with CoQ10 and TMZ significantly increased mRNA and protein expression of GR, 1.2-fold (*p* ≤ 0.05) and 1.8-fold (*p* ≤ 0.001), respectively (Figure 4(d)). Likewise, treatment with either CoQ10 or the combination increased *iNOS* mRNA expression 2.5-fold (*p* ≤ 0.05) or 3-fold (*p* ≤ 0.01), respectively, while iNOS protein expression remained unchanged (Figure 4(e)).

### 3.3. Single and Combined CoQ10 and TMZ Treatments Decreased the Invasiveness of RC6 Cells and Spheroids

**Figure 5:**
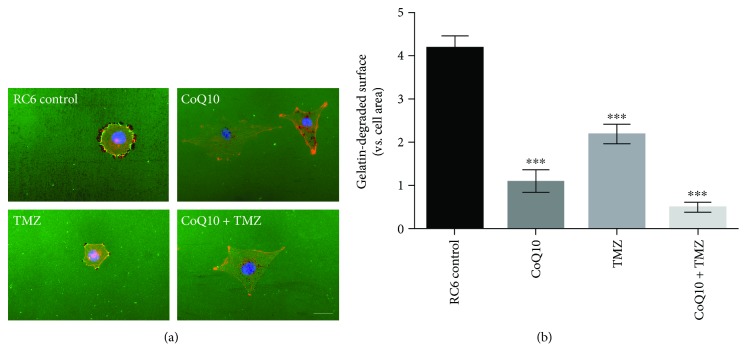
Suppression of gelatin degradation by RC6 cells. (a) Representative images of RC6 cells plated on Oregon Green® 488 Conjugate Gelatin (green) and treated with 10 *μ*M CoQ10, 250 *μ*M TMZ, or their combination. After 24 h, cells were stained with Hoechst 33342 (blue) and ActinRed 555 (red); dark areas represent spots of degraded gelatin. Scale bar = 200 *μ*m. (b) Histogram shows a degraded surface of Oregon Green® 488 Conjugate Gelatin per total area of cells in control and treatments. The average ±SEM obtained from three independent experiments is presented. Statistical significance is presented as *p* < 0.001 (∗∗∗).

Then, we tested the capacity of CoQ10 and TMZ to suppress gelatin degradation that was previously identified as an important feature of RC6 aggressive phenotype (Figure 5(a)). We found that the ability of RC6 cells to degrade gelatin was significantly inhibited (*p* ≤ 0.001) after single 10 *μ*M CoQ10 and 250 *μ*M TMZ treatments (2-fold and 4-fold, respectively), while their combination caused an 8-fold decrease in gelatin degradation compared to the untreated control (*p* ≤ 0.001) (Figure 5(b)).

**Figure 6:**
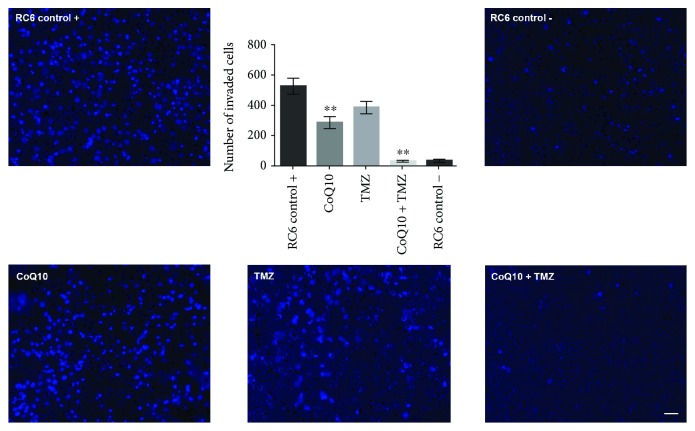
Anti-invasive potential of CoQ10, TMZ, and their combination. Representative images of RC6 cells that invaded through Matrigel to the opposite side of the membrane obtained after 24 h treatment. Nuclei of RC6 cells were stained with Hoechst 33342 (blue). “RC6 control +” is positive control (with FBS as chemoattractant) and “RC6 control –” is negative control (without chemoattractant). Scale bar = 50 *μ*m. Histogram shows the number of cells that invaded through Matrigel and passed the membrane. The average ±SEM obtained from three independent experiments is presented. Statistical significance is presented as *p* < 0.01 (∗∗).

Additionally, invasion assay was used to investigate the anti-invasive potential of 10 *μ*M CoQ10 and 250 *μ*M TMZ as well as their combination (Figure 6). We found that CoQ10 and its combination with TMZ significantly suppressed the invasion of RC6 cells through Matrigel by 50% and 80% (*p* ≤ 0.01), respectively, when compared to untreated cells.

**Figure 7:**
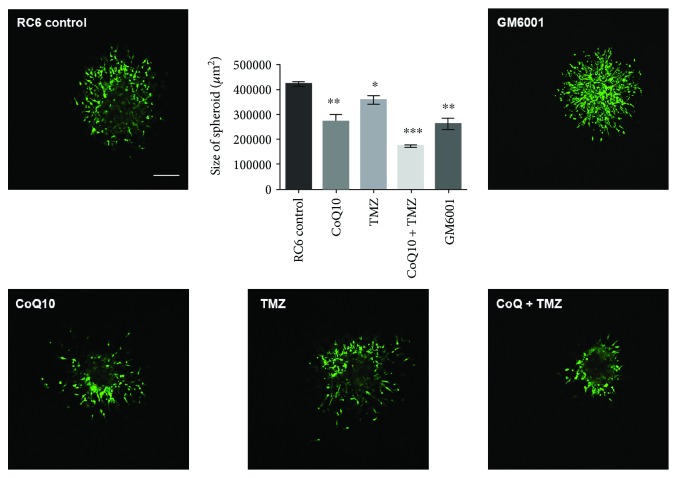
Effects of CoQ10, TMZ, and their combination on RC6 spheroid invasion. Representative images of RC6 spheroids embedded in collagen hydrogel and treated with 10 *μ*M CoQ10, 250 *μ*M TMZ, their combination, or 50 *μ*M GM6001. After 24 h, spheroids were stained with CAM (green) and PI (red). Scale bar = 200 *μ*m. Graph displays size of spheroids as a measure of their invasion potential. The average ±SEM obtained from three independent experiments is presented. Statistical significance is presented as *p* < 0.05 (∗), *p* < 0.01 (∗∗), and *p* < 0.001 (∗∗∗).

Finally, a 3D invasion assay was performed to evaluate the anti-invasive properties of the abovementioned treatments (Figure 7). Our results showed that CoQ10 significantly decreases the invasion of RC6 spheroids in collagen hydrogel by 33% (*p* ≤ 0.01), while the TMZ effect was weaker leading to a decrease of 20% (*p* ≤ 0.05). The most pronounced effect was observed in the combination treatment, which significantly inhibited invasion of RC6 spheroids by 60% (*p* ≤ 0.001). Furthermore, the effects of CoQ10, TMZ, and their combination on RC6 invasion ability were compared to the effect of GM6001, a broad-spectrum matrix metalloproteinase inhibitor. Treatment with 50 *μ*M GM6001 significantly decreased the invasion of RC6 spheroids by 38% (*p* ≤ 0.01), which was comparable to CoQ10 treatment.

**Figure 8:**
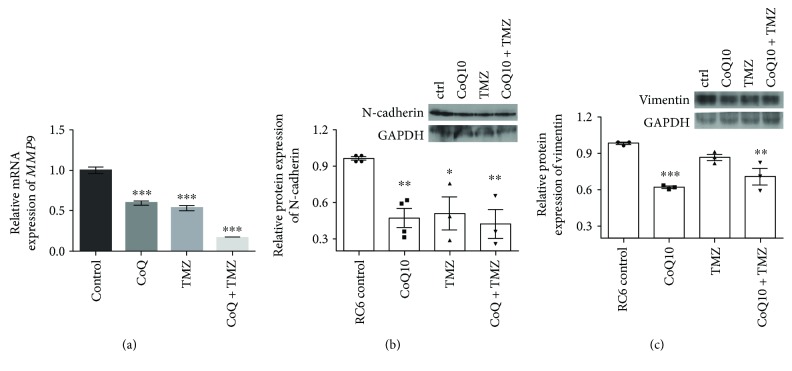
Effects of single and combined CoQ10 and TMZ treatments on MMP9 gene expression and protein expression of mesenchymal phenotype markers. Real-time qRT-PCR analysis was used to assess (a) MMP9 mRNA expression while Western blot analysis was used to determine (b) N-cadherin and (c) vimentin protein expression after 24 h treatment with 10 *μ*M CoQ10, 250 *μ*M TMZ, or their combination. The mRNA expression and protein expression were normalized to the internal control, *β*-actin and GAPDH, respectively. Relative protein expression is accompanied by representative immunoblots. The average ±SEM was obtained from at least three independent experiments. Statistical significance is presented as *p* < 0.05 (∗), *p* < 0.01 (∗∗), and *p* < 0.001 (∗∗∗).

Further, we searched for the mechanism responsible for the anti-invasive potential of CoQ10 and TMZ combination. To that end, we analyzed *MMP9* mRNA expression by qRT-PCR as well as N-cadherin and vimentin protein expression by Western blot (Figure 8). CoQ10, TMZ, and their combination significantly decreased the expression of *MMP9* mRNA in RC6 cells by 1.7-fold, 1.9-fold, and 6-fold, respectively (*p* ≤ 0.001, Figure 8(a)) indicating a strong potentiation of the studied effect when CoQ10 and TMZ were applied in combination. We found that CoQ10, TMZ, and their combination induced a similar reduction in N-cadherin expression 2-fold (*p* ≤ 0.01), 1.9-fold (*p* ≤ 0.05), and 2.3-fold (*p* ≤ 0.01), respectively (Figure 8(b)), while CoQ10 and the combination led to a significant decrease of vimentin expression in RC6 cells by 1.6-fold (*p* ≤ 0.001) and 1.4-fold (*p* ≤ 0.01), respectively (Figure 8(c)).

### 3.4. Anti-Invasive Effects of Single and Combined CoQ10 and TMZ Treatments of RC6 Orthotopic Allograft

**Figure 9:**
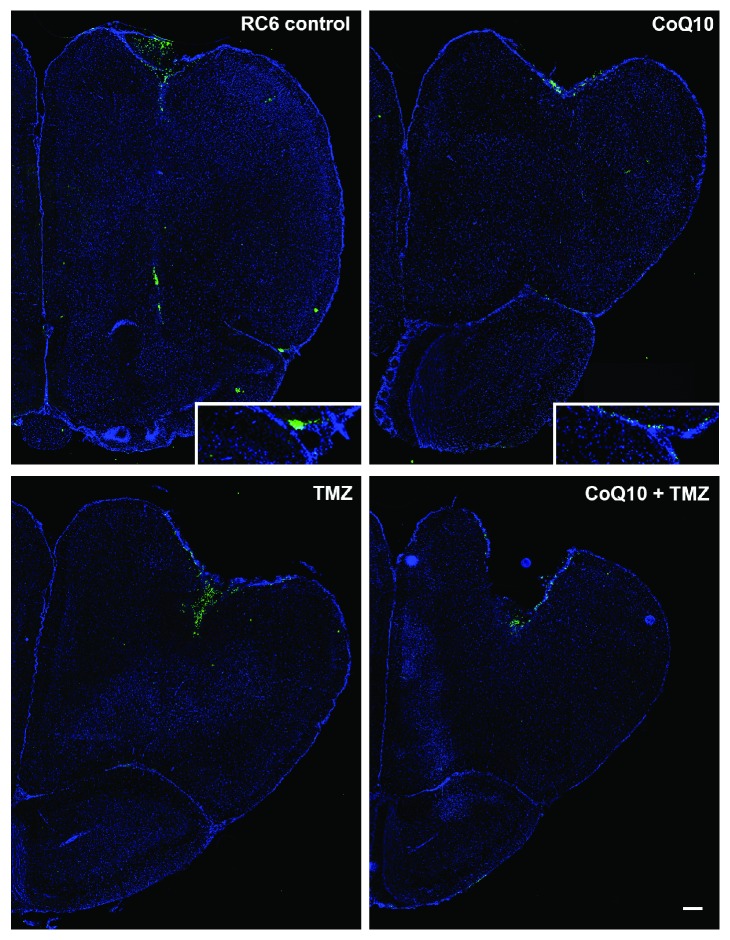
*In vivo* study of RC6 invasion in orthotopic allograft. Representative images of Wistar rat brain coronal sections from the RC6 control group, CoQ10 group, TMZ group, and CoQ10 + TMZ group. Inoculated cells were stained with fluorescent dye CFSE (green), and nuclei were counterstained with Hoechst 33342 (blue). Scale bar = 200 *μ*m. Enlarged images in the corners of RC6 control and CoQ10 group images display RC6 cells at the edge of and in the ipsilateral olfactory bulb.

Motivated by the results obtained *in vitro*, our further research was aimed at confirming these results *in vivo*. Previously, we had developed an orthotopic allograft by inoculation of CFSE fluorescently labeled RC6 cells in the Wistar rat brains. To study the effects of CoQ10, TMZ, and their combination, we divided animals into 4 groups: the control group, group treated with 10 mg/kg CoQ10 (i.p., twice a week for three weeks), the group treated with 4 mg/kg TMZ (i.p., 5 times consecutively during the second week), and the group treated with the combination of CoQ10 and TMZ (i.p., scheduled as single treatments). Coronal brain sections showed that RC6 cells in the control group migrated into distant ipsilateral brain structures and were found as far as in the ipsilateral olfactory bulb. Cells were present in dispersed clusters representing one of the main features of their infiltration pattern. Treatment with CoQ10 exhibited an anti-invasive effect as seen on corresponding coronal brain sections. RC6 cells were found in clusters near the injection site and at the outermost edge of the ipsilateral olfactory bulb. Fluorescently labeled RC6 cells on coronal brain sections of animals treated with TMZ were detected in distant brain regions where they followed an already established invasion pattern. The most noticeable anti-invasive effect was observed in animals treated with CoQ10 and TMZ combination, where the majority of RC6 cells stayed near the injection site (Figure 9).

## 4. Discussion

Despite the growing knowledge of the GBM development and progression, treatment advances and options are still poor. Results of current treatments for newly diagnosed and recurrent GBM are disappointing, and there is an urgent need for finding new modalities in chemotherapy. Several studies were undertaken to increase TMZ efficacy in GBM. Thus, various substances including interferon-*β* [30], ribonucleotide reductase inhibitors [31], and valproic acid [32] increased the sensitivity of GBM to TMZ through different mechanisms.

In this study, we used a TMZ-resistant RC6 cell line in order to test the potential of CoQ10, as an antioxidant, to enhance TMZ efficacy. CoQ10 drew our interest since it is a natural compound also synthesized by human cells [33]. In addition, it is a lipophilic compound able to cross the blood-brain barrier [34], which makes it suitable for glioma treatment. Due to its redox modulatory potential, CoQ10 may influence invasiveness and resistance, the key features associated with GBM pathology. Indeed, we showed that CoQ10 increased the sensitivity of RC6 cells to TMZ that is in accordance with a recent study in which CoQ10 increased radio- and TMZ sensitivity in human GBM cell lines, without affecting normal astrocytes [22]. However, 2D cell cultures are not able to portray the complex cellular interactions and specific environmental settings occurring in tumors, which is often the reason for drug failure in later research stages. Therefore, we used a 3D microfluidic model to closely mimic microenvironmental conditions and their effect on drug response [35]. Within the microfluidic device, RC6 cells embedded in collagen hydrogel in the central microchamber represented a “tumor slice” with specific microenvironmental conditions at different distances from surrogate blood vessels (the lateral microchannels). For this one and all subsequent experiments, we used 10 *μ*M CoQ10, the concentration detected in human plasma after oral ingestion [36]. The results obtained using the 3D model confirmed that CoQ10 and TMZ combination was more effective in inducing RC6 cytotoxicity than TMZ treatment alone. Combined treatment created wider and more intense areas of cell death around the lateral microchannels probably due to different spatial distributions.

Oxidative stress and ROS have an important role in EMT through regulation of signaling pathways (e.g., NF-*κ*B and TGF-*β*) required for MMP activation, ECM degradation, and loss of cell-cell junctions, as well as reversible or irreversible oxidative modification of the cytoskeleton protein components [37]. Increased ROS levels can activate NF-*κ*B signaling and thus induce transcription of vimentin and MMPs as well as EMT-related morphological changes [38, 39]. Therefore, ROS reduction may diminish EMT progression implying antioxidants as beneficial agents for the inhibition of cancer invasion and metastasis.

Exogenously administered CoQ10 predominantly incorporates into mitochondrial membranes [40]. A recent study showed that incubation of human GBM cells with CoQ10 reduced mitochondrial O_2_^−^ production and H_2_O_2_ levels [22]. Our results in a 3D microfluidic device model confirmed that CoQ10 alone reduced ROS production in rat RC6 cells causing a significant increase in MnSOD and CAT protein expression. Combined treatment with CoQ10 and TMZ led to a significant increase in MnSOD, CuZnSOD, CAT, and GR protein expression. Although CoQ10 alone and cotreatment with TMZ prominently increased *iNOS* mRNA expression, this change was not reflected at the protein level. In this way, CoQ10 exerted its antioxidant activity in RC6 cells by direct scavenging of ROS (e.g., O_2_^−^), the transformation of O_2_^−^ to H_2_O_2_, and its subsequent degradation to H_2_O as a result of an increase in mitochondrial antioxidant capacity.

According to Tsai et al., overexpression of catalase leads to the decreased production of H_2_O_2_ and consequent inhibition of lung cancer cell migration and invasion [41]. Other evidences have established a positive correlation between H_2_O_2_ content and MMP2 activity in MCF-7 breast cancer cells also showing that CoQ10 application decreases H_2_O_2_ production leading to the inhibition of MMP2 activity [42]. Likewise, CoQ10 caused a decrease in O_2_^−^ level in mitochondria, inhibited MMP2 and MMP9 activity, and reduced tumor volume and the number of metastasis in mouse lung carcinoma [43]. We demonstrated that combined treatment with CoQ10 and TMZ more efficiently decreased gelatin degradation and Matrigel and spheroid invasion in comparison with single treatments. Moreover, galardin (GM6001), a potent broad-spectrum MMP inhibitor, exhibited reduction of RC6 spheroid invasion at the level equivalent to single CoQ10 treatment.

Significant inhibition of *MMP9* gene expression, a dominant form of gelatinase expressed in RC6 cells, together with significant inhibition of N-cadherin and vimentin, mesenchymal phenotype markers, implied both MMPs and EMT blockage as a possible anti-invasive mechanism of combined CoQ10 and TMZ treatment. Other potent antioxidants, such as resveratrol and curcumin, increased the expression of epithelial phenotype marker E-cadherin and reduced the expression of mesenchymal phenotype markers, vimentin and fibronectin, thus suppressing TGF-*β*1-induced EMT in lung and hepatoma cell lines [44, 45]. Our *in vitro* results were confirmed in RC6 orthotopic allograft, where CoQ10 and TMZ cotreatment efficiently interrupted the distinctive infiltration pattern of RC6 cells towards the ipsilateral olfactory bulb. Other authors also showed that CoQ10 could be valuable in combination with clinically approved chemotherapeutics, i.e., CoQ10 combined with tamoxifen significantly reduced the tumor weight and volume in rat mammary carcinoma [46].

## 5. Conclusions

In summary, we have demonstrated that CoQ10 sensitizes RC6 cells to TMZ and enhances TMZ-induced cell death in a 3D model. CoQ10 and TMZ applied in combination inhibited invasion of RC6 cells *in vitro* and *in vivo*, by modifying mitochondrial antioxidant capacity and expression of MMP9 and EMT markers. Significant advances in GBM therapy have not yet been achieved despite invested efforts. Recent clinical studies of erlotinib and cilengitide, as promising targeted therapeutics, in combination with standard GBM treatments have failed in phase II or III clinical trials [47, 48]. Our study highlights the benefit of CoQ10 supplementation in GBM therapy and provides a solid basis for further preclinical and clinical research of CoQ10 and TMZ combination. Therefore, CoQ10 which is widely used in human diet and different pathologies as a supplement could be considered as a valuable addition to standard GBM treatment.

## Data Availability

The rough data and analyses used to support the findings of this study are available from the corresponding author upon request.

## Abbreviations

GBM: Glioblastoma
TMZ: Temozolomide
ECM: Extracellular matrix
EMT: Epithelial-mesenchymal transition
MMP: Matrix metalloproteinase
TIMP: Tissue inhibitors of metalloproteinases
uPA: Urokinase-type plasminogen activator
BCNU: 1,3-Bis(2-chloroethyl)-1-nitrosourea
ROS: Reactive oxygen species
MnSOD: Manganese superoxide dismutase
CuZnSOD: Copper/zinc superoxide dismutase
CAT: Catalase
GPx: Glutathione peroxidase
GR: Glutathione reductase
iNOS: Inducible nitric oxide synthase
CoQ10: Coenzyme Q10
DMSO: Dimethyl sulfoxide
FBS: Fetal bovine serum
EDTA: Ethylenediaminetetraacetic acid
SRB: Sulforhodamine B
CI: Combination index
CAM: Calcein
PI: Propidium iodide
SDS-PAGE: Sodium dodecyl sulfate polyacrylamide gel electrophoresis
GAPDH: Glyceraldehyde 3-phosphate dehydrogenase
HRP: Horseradish peroxidase
PFA: Paraformaldehyde
CFSE: Carboxyfluorescein succinimidyl ester
i.p.: Intraperitoneal
NF-*κ*B: Nuclear factor kappa B
TGF-*β*: Transforming growth factor beta.
